# Prognostic factors in uterine adenosarcoma: subanalysis of the SARCUT study

**DOI:** 10.3389/fonc.2024.1393707

**Published:** 2024-05-21

**Authors:** Rosanna Mancari, Yildirim Yusuf, Ronalds Macuks, Patriciu Achimas-Cadariu, Jurgen Martinus Piek, Isabella Sperduti, Giacomo Corrado, Enrico Vizza, Ignacio Zapardiel

**Affiliations:** ^1^ Gynecologic Oncology Unit, IRCCS Regina Elena National Cancer Institute, Rome, Italy; ^2^ Gynecologic Oncology Department, Tepecik Training and Research Hospital, Izmir, Türkiye; ^3^ Latvian Oncology Center of Riga Eastern Clinical University Hospital, Riga, Latvia; ^4^ Gynecologic Oncology Department, Institute of Oncology Prof. Dr. I Chiricuta, Cluj-Napoca, Romania; ^5^ Gynecologic Oncology Department, University of Medicine and Pharmacy Iuliu Hatieganu, Cluj Napoca, Romania; ^6^ Gynecologic Oncology Department, Catharina Ziekenhuis Department of Gynecology, Eindhoven, Netherlands; ^7^ Unit of Biostatistical, IRCCS Regina Elena National Cancer Institute, Rome, Italy; ^8^ Gynecologic Oncology Unit, Department of Woman, Child and Public Health, Fondazione Policlinico Uni-versitario A. Gemelli IRCCS, Rome, Italy; ^9^ Gynecologic Oncology Unit, La Paz University Hospital, Madrid, Spain

**Keywords:** adenosarcoma, uterine neoplasms, prognosis, disease-free survival, survival rate

## Abstract

**Objective:**

The purpose of the study was to analyse the role of prognostic factors on the risk of recurrence and overall survival of patients with uterine adenosarcoma.

**Methods:**

A retrospective international multicentre study involving 46 centres collected 32 cases of uterine adenosarcoma, and these cases were included in the present subanalysis. Clinical and demographic features and tumour characteristics were gathered, as well as information on treatment and relapse. Disease-free and overall survival were analysed.

**Results:**

The 5-year disease-free survival (DFS) was 85.3% and the 5-year overall survival (OS) rate was 89.5%. The risk factors significantly associated with overall survival were age (HR 1.09, 95% CI 1.03–1.15; *p* = 0.004) and FIGO stage II–III (HR 17.75, 95% CI 2.87–109.93; *p* = 0.002). Patients who experienced early relapse (within 12 months) had a tumour size >30 mm and advanced stage. The majority of recurred cases were treated with radiotherapy or surgery and obtained a good response rate.

**Conclusion:**

The most significant prognostic factors in uterine adenosarcoma were age and FIGO stage and, indirectly, tumour size at diagnosis. The use of secondary surgery and/or radiotherapy could help in prolonging the disease-free status of the patients.

## 1 Introduction

Uterine adenosarcoma is a rare tumour accounting for 5.5%–9% of all uterine sarcomas and 0.2% of all uterine neoplasms (1). Patients with adenosarcoma are usually diagnosed with stage I disease and have a 5-year overall survival (OS) of 60%–80% (1). First described by Clement and Scully in 1974 (2), this neoplasia consists of a malignant stromal component and benign epithelium (3, 4). The stromal component is typically characterised by a low-grade spindle cell sarcoma, while the epithelial components are commonly endometrioid exhibiting frequent metaplasia and occasionally atypia (4).

While the sarcomatous component typically stains for CD10, the epithelial part regularly exhibits estrogen and progesterone receptors (5).

Uterine adenosarcoma (AS) is the intermediate step of the spectrum beginning with benign entities such as adenofibromas and adenomyomatosis and ending in the high end of the spectrum with carcinosarcoma (6).

It usually manifests as a polypoid endometrial tumour, but it can be found in the cervix, ovary or other extrauterine areas.

It has the highest incidence in the fifth and sixth decades, but it may be diagnosed at a younger age (3–8). AS is usually defined as a low-grade sarcoma (3), and it shows wide variability in survival rate, depending on the possible manifestation of some negative prognostic factors, mainly on the presence of the sarcomatous overgrowth (SO)—when more than 25% of the tumour consists of a high-grade sarcomatous component—which is associated with worse prognosis (3). The variant with SO exhibits aggressive behaviour along with frequent recurrences and shorter survival (3).

Additional known prognostic factors include stromal atypia, deep myometrial invasion and vascular space invasion, which are associated with aggressive evolution and lend the disease the name of high-grade adenosarcoma (9). The presence of hormone receptors, CD10 and WT1 expression is generally associated with a good prognosis and contributes to the diagnosis of the so-called low-grade adenosarcoma (10).

The standard treatment of uterine adenosarcoma consists of performing a total hysterectomy, with or without bilateral salpingo-oophorectomy, and the removal of all visible disease (1). Adjuvant treatment is controversial due to a lack of prospective trials. In the same way, the optimal treatment for relapsed uterine adenosarcomas is yet to be clearly defined: radiotherapy, chemotherapy and hormonal therapy may be potential options (11).

Identifying the most relevant prognostic factors is essential in order to improve the outcomes and proper management of patients with this diagnosis.

The aim of this study was to analyse the prognostic factors associated with oncological outcomes in patients diagnosed with uterine adenosarcoma via subanalysis of the patients included in the multicentric international SARCUT study.

## 2 Materials and methods

The SARCUT (SARComa of the UTerus) study was a retrospective international multicentre study of patients diagnosed with uterine sarcoma. This study involved 46 different European institutions which included 966 patients diagnosed with uterine sarcoma between January 2001 and December 2007, and patients were followed until 2012. For the current subanalysis, we selected patients diagnosed with uterine adenosarcoma, including 32 cases from 18 participating institutions.

The inclusion criteria for the present analysis included a pathological diagnosis of uterine AS, as well as primary surgical treatment performed at the participating centre. The review from the Institutional Review Board (IRB) was first obtained at the coordinating centre (#PI-1382), and for the rest of the participating institutions, approval was obtained according to local regulation requirements. After receiving approval from the IRB, informed consent was not necessary to review the participants’ medical records. Uterine AS was classified according to the 2009 International Federation of Gynecology and Obstetrics (FIGO) classification system (12). The baseline clinical and demographic features of the patients and the tumour characteristics of uterine adenosarcoma were collected.

Primary treatment and recurrences were recorded; the terminology for surgical procedures, the sites of recurrence and adjuvant treatment were standardised. Surgical procedures were adapted to address the extension of disease, and complete cytoreduction was achieved when no residual tumour was present after surgery (13). Adjuvant treatment was administered at the institution’s discretion based on local protocols.

After primary treatment, follow-up was performed every 3 months on average for the first year, every 6 months until the fifth year and then annually. Patients lacking follow-up information were excluded from the analysis. Imaging techniques involved an ultrasound of the abdomen, alternating with either computed tomography or magnetic resonance imaging, depending on clinical practice and availability at each centre.

Disease-free survival (DFS) was defined as the interval between the end of primary treatment and the appearance of recurrence. Recurrence was defined as the appearance of the tumour after primary treatment, during the follow-up period. We distinguished between pelvic and distant recurrences based on the site of the relapsed disease. Secondary surgery, hormonal therapy, radiotherapy and/or chemotherapy were administered at the institution’s discretion in accordance with local protocols. OS was defined as the length of time from the date of diagnosis to the death or last follow-up.

Data collection was conducted via a web-based database. Individual researchers were responsible for the registration of their own cases. This manuscript was prepared in accordance with STROBE guidelines (14).

### 2.1 Statistical analysis

Descriptive statistics were used to describe patient characteristics. Continuous variables are presented as median and range. Discrete variables are presented as absolute values and percentages. The chi-square test or Fisher’s exact test was used to estimate the association between categorical variables when appropriate.

Overall survival and DFS were calculated by the Kaplan–Meier survival curves from the date of the surgery until relapse or demise. If a patient were alive, survival was then censored at the time of the last visit. DFS was calculated from the date of surgery to the date of relapse or death. If a patient had not relapsed, DFS was censored at the time of the last visit. The log-rank test was used to assess differences between subgroups. Significance was defined at the *p <*0.05 level. The hazard risk and the confidence limits were estimated for each variable using the Cox univariate model. A multivariate Cox proportional hazard model was also developed using stepwise regression (forward selection) with variables, which were significant in the univariate analyses. The enter limit and remove limit were *p* = 0.10 and *p* = 0.15, respectively. The statistical software used was SPSS v.21 (SPSS Inc., Chicago, IL, USA).

## 3 Results

A total of 32 women with uterine AS were identified. The baseline clinical data and demographics of the study population are illustrated in **Table 1**.

**Table 1:** Baseline characteristics of the patients with uterine adenosarcoma.

Variables	Number of cases (%)
Age, years (median, range)	61.5 (17–88)
Menopause	22 (68)
Smokers	2 (6.2)
Parity, births (median, range)	2 (0–4)
Previous fibroids	1 (3.1)
Previous pelvic radiation	0
Previous use of tamoxifen	1 (3.1)
Symptoms	
Pain	0
Pelvic mass	0
Bleeding	27 (84.4)
None	5 (15.6)
FIGO stage	
I	25 (78.2)
II	4 (12.5)
III	3 (9.3)
IV	0
Margin involvement	1 (3.1)
Tumour size, mm (median, range)	30 (2–170)
Extrauterine involvement	3 (9.3)
Surgical approach	
Laparoscopy	4 (12.5)
Laparotomy	27 (84.4)
Vaginal	1 (3.1)
Surgical procedure	
Hysterectomy	32 (100)
Bilateral salpingo-oophorectomy	29 (90.6)
Omentectomy	2 (6.2)
Pelvic lymphadenectomy	7 (21.8)
Para-aortic lymphadenectomy	1 (3.1)
Appendectomy	1 (3.1)
Involved margin after resection	
No	31 (96.9)
Yes	1 (3.1)
Radiotherapy	13 (40.6)
Chemotherapy	2 (6.2)

The median age at diagnosis was 61.5 (range 17–88) years and 22 (68%) patients were postmenopausal at the time of diagnosis. The unique reported symptom was abnormal vaginal bleeding, present in 27 (84.3%) patients, while 5 (15.6%) women were asymptomatic. The tumour was diagnosed after undergoing a routine exam in addition to detecting an enlarged uterus. Twenty-five (78.1%) patients had FIGO stage I, 4 (12.5%) patients presented FIGO stage II and 3 (9.4%) patients had FIGO stage III. Among the three FIGO stage III cases, two of them presented pathological confirmation of nodal metastases, and one patient presented pathologic enlarged nodes at the preoperative CT scan with suspected vascular infiltration; therefore, no surgery was carried out. This last patient received radiotherapy with complete response and remained free of disease for 79 months.

The median tumour size (referred to as the main nodule at diagnosis) was 30 (range 2–170) mm. Lymphovascular space invasion (LVSI) determination was available in only 12 (37.5%) patients and was always reported as negative. Sarcomatous overgrowth was available in only three cases; therefore, we decided not to consider it in the statistical analysis.

Concerning surgical treatment, all patients underwent a total hysterectomy. The surgical route taken was laparoscopy in 4 (12.5%) cases, laparotomy in 27 (84.4%) cases and vaginal in 1 (3.1%) case. Twenty-nine (90.6%) patients also underwent a bilateral salpingo-oophorectomy (BSO). Three (9.4%) patients received additional procedures, such as omentectomy in two cases and appendectomy in one case. Complete cytoreduction was achieved in 31 (96.9%) patients. Seven (21.9%) patients underwent pelvic lymphadenectomy, one of them with an additional para-aortic dissection.

Adjuvant treatment was administered in 15 (46.8%) cases, radiotherapy in 13 (40.6%) patients and chemotherapy in 2 (6.2%) patients.

**Figure 1:**
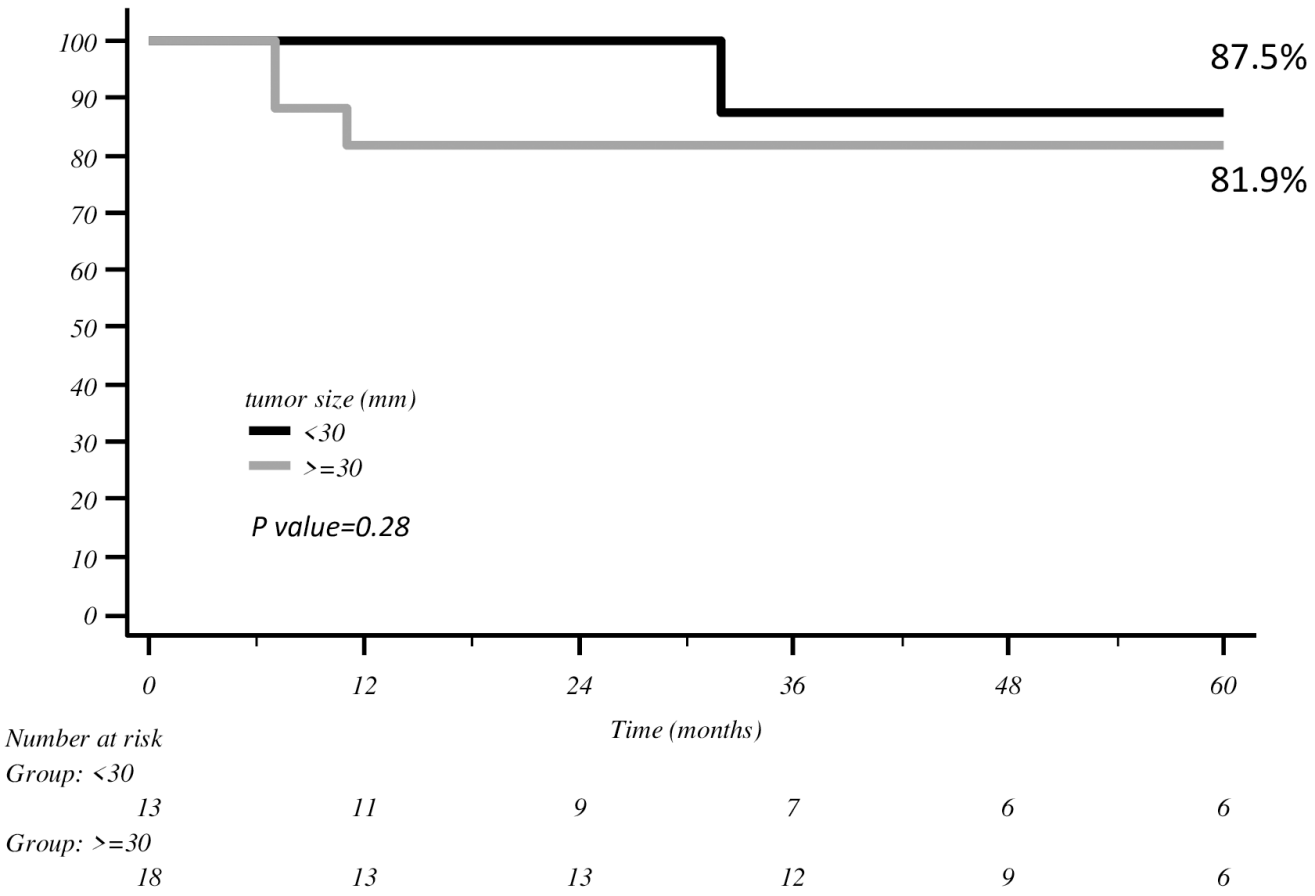
DFS with Kaplan–Meier curve according to tumour size.

At a median follow-up of 48 months (range 1–140), the 5-year DFS was 85.3% and the 5-year OS was 89.5%. Five (15.6%) patients relapsed, two (6.25%) with pelvic recurrence, two (6.25%) with pelvic and distant disease and one (3.1%) with distant metastasis only. Three (9.4%) of them relapsed within 1 year of follow-up. When comparing the 5-year DFS according to tumour size, we did not find any significant differences between patients with tumours measuring <30 mm vs. ≥30 mm (87.5% vs. 81.9%, respectively; *p* = 0.28) (**Figure 1**).

**Figure 2:**
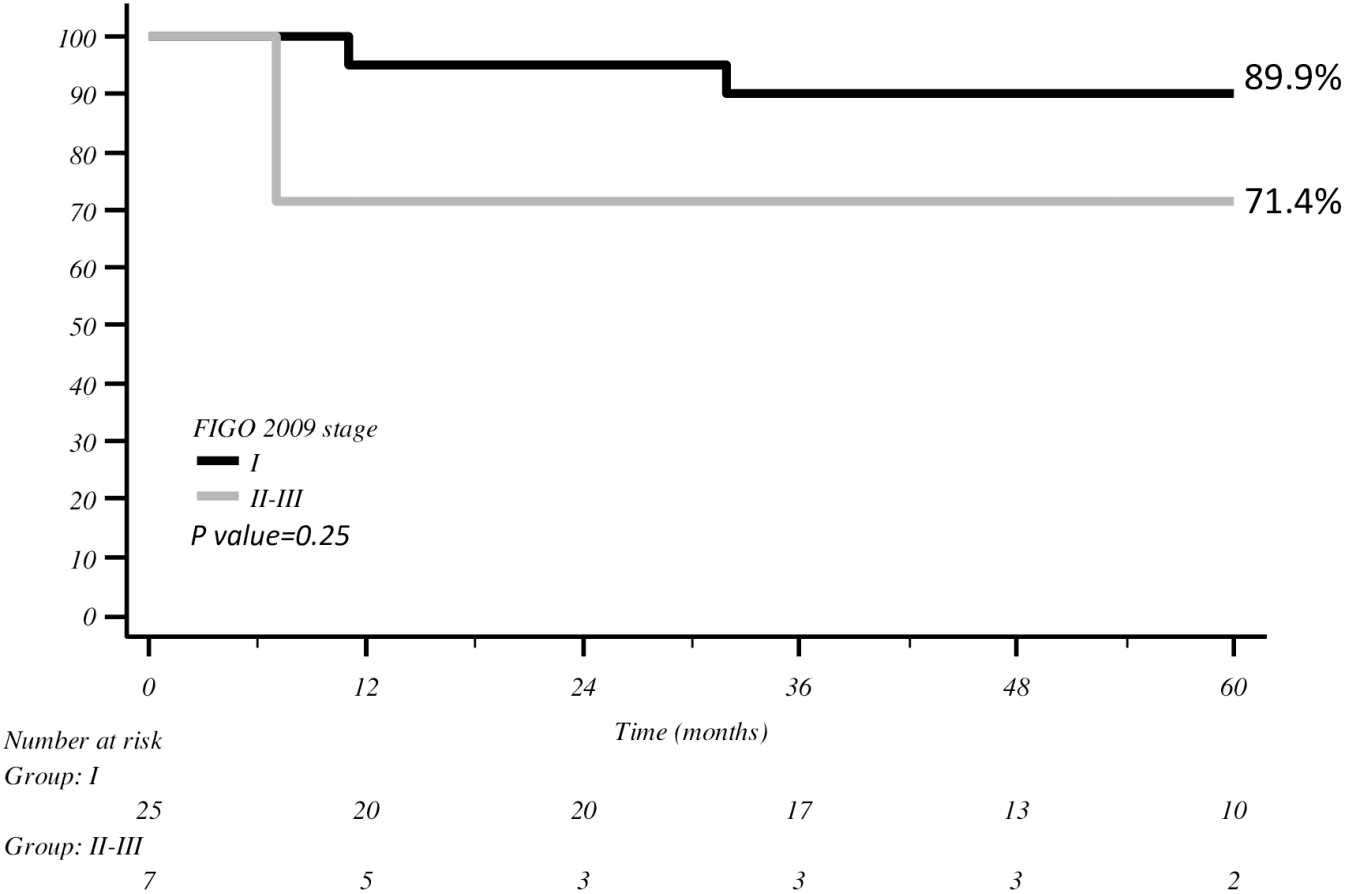
DFS with Kaplan–Meier curve according to FIGO stage.

The same findings were observed between FIGO stage I vs. II–III (89.9% vs. 71.4%, respectively; *p* = 0.25) (**Figure 2**).

**Figure 3:**
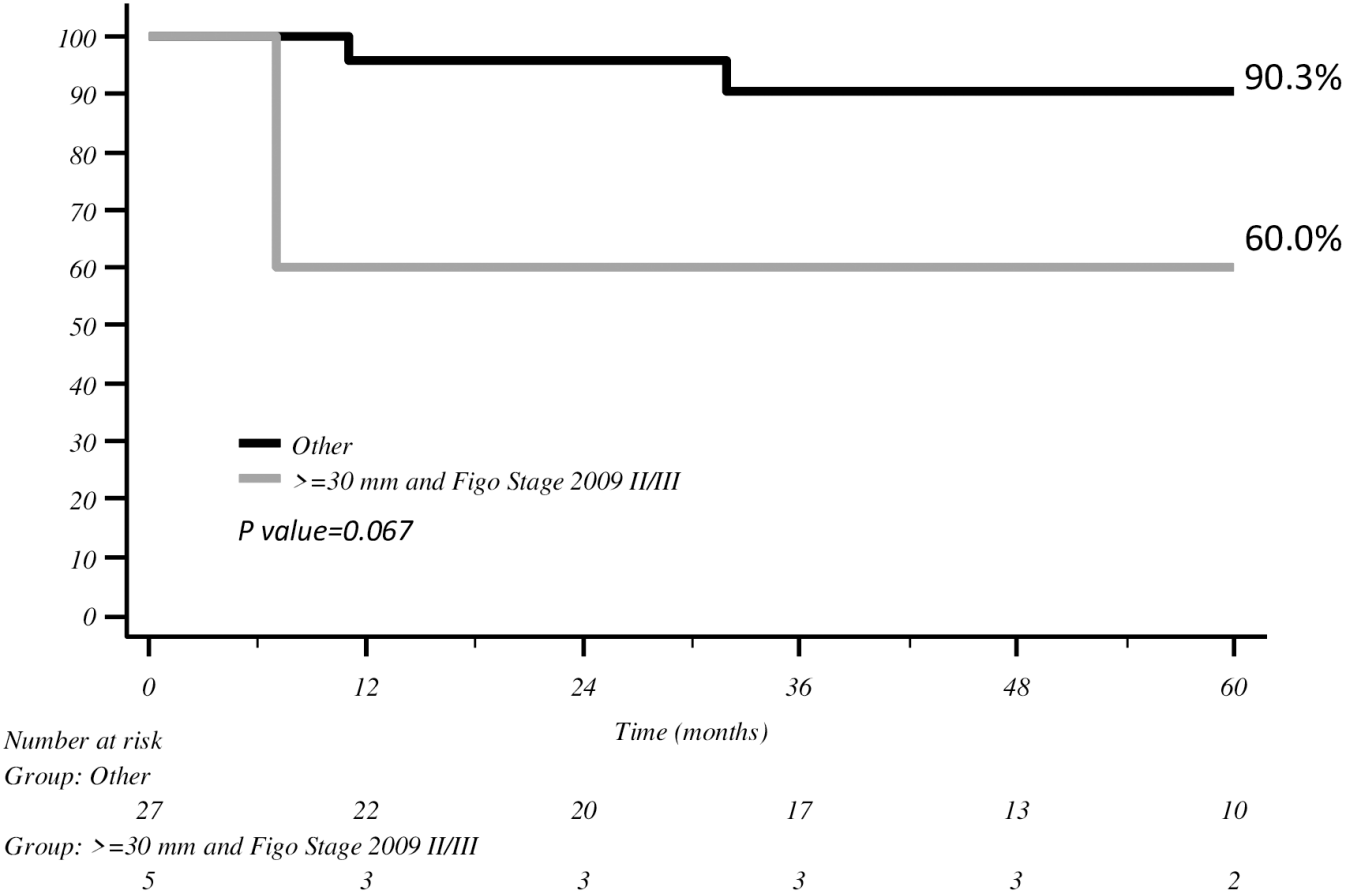
DFS with Kaplan–Meier curve according to tumour size >30 mm and FIGO stage II–III.

Both tumour size ≥30 mm and FIGO stage II–III indicated a trend towards worse prognosis compared with the rest of the population with a 5-year DFS of 60% (*p* = 0.06) (**Figure 3**).

In the multivariate analysis, the only risk factors significantly associated with overall survival were age (HR 1.09, 95% CI 1.03–1.15; *p* = 0.004) and FIGO stage II–III (HR 17.75, 95% CI 2.87–109.93; *p* = 0.002).

When we analysed patients with early relapse (within 12 months), we observed that they had a tumour size >30 mm and at an advanced stage more frequently than others; however, this was deemed not significant.

Relapsed patients were treated with surgery or radiotherapy. Two (6.2%) patients with early relapse survived for 45 months and 17 months after secondary treatment, and the third one was lost to follow-up.

The patient relapsed with only distant metastasis after 66 months and was subsequently treated with secondary surgery and obtained a DFS of 95 months. Only one (3.1%) patient died from AS, and two more patients passed away from other diseases.

## 4 Discussion

This study evaluated the prognostic factors impacting the oncological outcome of patients with uterine adenosarcoma. The most important prognostic factors seemed to be age and FIGO stage at diagnosis. In addition, we observed a long 5-year DFS and OS, where our results were found to be consistent with the most favourable cases reported in the literature.

Carroll found similar results in his publication, where older age and FIGO stage were associated with worse PFS on multivariate analysis, in addition to sarcomatous overgrowth and LVSI (15). In a huge study by Arend, 544 patients with uterine AS were identified and their 5-year survival was 79% for stage I disease and 48% for stage III disease (16), confirming the importance of FIGO stage in the prognosis of these women. Similarly, Zaloudek found that extrauterine spread is one of the most relevant prognostic factors (17), and Ulrich upheld the good prognosis of patients with stage I disease (18).

Carroll (15) examined 74 patients with uterine AS, showing a worse survival with increasing age. Nathenson described a significant worse prognosis in terms of DFS and OS when patients were older than 53 years (19). These data confirm that age is a significant prognostic factor in women with uterine AS like in many other neoplastic diseases.

Despite the good prognosis of the disease, three out of our five patients with relapse recurred within 1 year (early relapse, ER). Therefore, we decided to concentrate on this group and observed that these patients exhibited large tumour size and advanced FIGO stage more frequently than late or non-relapsed patients. Even if we did not find any statistically significant differences, we observed a trend of significant to worse prognosis among patients with both tumour size ≥30 mm and FIGO stage II–III, which partially agrees with the results of the multivariate analysis performed.

The high proportion of pelvic recurrences in the group of ER patients could be related to local factors, such as myometrial invasion and tumour size, although we cannot assess it due to the number of cases. Even though we could not find any significant differences in the multivariate analysis, these data are congruent with the literature, where size is considered a prognostic factor (20) even if its power is weaker than that of sarcomatous overgrowth, myometrial invasion and lymphovascular invasion. Seagle in 2016 affirmed that increasing tumour size was associated with a decline in OS in patients with adenosarcoma (20). In his series, the risk of death was related to an increase in each centimetre in tumour size among patients with uterine AS. Machida found that patients with uterine AS have smaller-sized tumours than those diagnosed with endometrial stromal sarcoma or uterine leiomyosarcoma, both presenting worse prognosis (21). In this setting, the use of adjuvant radiotherapy could be justified to prevent local recurrence (22), but some retrospective trials failed to demonstrate its efficacy since it had no effect on survival for women with uterine AS (16, 21, 23). Moreover, to our knowledge, no prospective trials have been planned due to the rarity of the disease. In another series, adjuvant radiotherapy has been shown to be a negative prognostic factor, but this was probably due to selection bias, in relation to the high percentage of risk factors in that group of women (20).

According to the literature, the most important prognostic factors for uterine AS are age, myometrial invasion, sarcomatous overgrowth, presence of LVSI and node involvement. In addition, tumour size, mitotic index and tumour necrosis could have an impact but cannot be considered independent prognostic factors (6, 22). During the M.D. Anderson Cancer experience, the median tumour size of AS ranged from 60 mm to 200 mm (22); our median tumour size was much lower (30 mm), and this fact could be considered a bias, but it can also justify the better prognosis of our patients.

In the GOG study published in 1992 (6), 31 patients with stage I–II uterine AS were enrolled with a median age of 62 years, similar to our population. In 85% of patients, the diameter of the uterine lesion ranged between 2 cm and 10 cm. This study underlined the importance of SO in worsening the prognosis of patients and the tendency towards the association between older age and worse prognosis, data confirmed by many other studies (22–24). The population with SO consisted of 17 patients (55%) and was compared to a population without SO: the mean age of the first group was approximately 5 years older than the second, even if it was not statistically significant. Recurrence occurred in 44% of the first group and in 14% of the second one, and 31% of the patients died in the first group vs. only 7% of the second one. Denschlag found that mortality for typical adenosarcoma was 10%–25 %, while the mortality rate for adenosarcoma with sarcomatous overgrowth was up to 75 % (25).

Several publications illustrated a low rate of nodal metastases among patients with uterine AS, ranging from 3% to 6.5% (6, 15, 16). In a SEER database analysis, 994 cases with uterine AS were identified, and the incidence of lymph node metastasis was 2.9% (21). For this reason, the role of lymphadenectomy in women with uterine AS appears to be limited similar to patients with uterine leiomyosarcoma, and lymphadenectomy is not routinely undertaken (15).

Nevertheless, lymph node involvement may be an indicator of a poorer prognosis; therefore, it is suggested to first try all options to achieve optimal surgical debulking, thus promoting the use of lymphadenectomy when bulky nodal disease is present (22, 25).

Recently, another histologic pattern has been described called the high-grade stromal component (26). It is defined by the presence of severe nuclear pleomorphism and is usually but not exclusively present in the context of sarcomatous overgrowth. We could not analyse the SO nor the high-grade stromal component in our group of patients since these characteristics were not collected nor included in our data.

One of our study’s main weaknesses is the paucity of data regarding some prognostic factors, such as myometrial invasion and sarcomatous overgrowth, as well as the low number of cases. Conversely, one of our study’s main strengths includes the long period of follow-up and the high number of involved participating centres.

It is worth highlighting the importance of FIGO stage and, indirectly, tumour size at diagnosis, which will impact the prognosis of the disease.

The role of systemic therapies in uterine AS has yet to be well established. Due to a lack of clinical trials for uterine AS, there is no standard role of adjuvant chemotherapy for this disease (1), and patients at low risk of recurrence should be thoroughly observed without undergoing any adjuvant therapy.

Guidozzi described the history of three patients with stage II–III uterine AS, who received neoadjuvant chemotherapy and surgery in two cases and surgery followed by chemotherapy and radiotherapy in the third case, obtaining a long disease-free interval, ranging from 34 months to 56 months of follow-up (27).

Verschraegen published his series and described partial response and stable disease after chemotherapy in approximately 50% of patients in recurrent patients (22). These data could reinforce the significance of systemic therapy, even if the number of patients was small and there were scant data on the other prognostic factors.

It is important to also underline the role of secondary therapies, both radiotherapy and secondary surgery, that allowed our relapsed patients to maintain a disease-free status for a relatively long period of time.

In Carroll’s paper, the median survival time from the date of recurrence in women who underwent a secondary cytoreduction for relapse was 16.1 months, compared with 8.4 months in patients who did not undergo secondary surgery (15). In reading the results by Krivak, we can hypothesise a correlation between the site of recurrence and the prognosis of the patient since distant relapses—mainly pulmonary—were associated with shorter survival. This is probably due to the difficulties in cytoreducing some areas more than others, consequently highlighting the importance of secondary surgery (28).

In the literature, doxorubicin-based regimens and gemcitabine/docetaxel chemotherapy have been reported to be useful in recurrent and/or metastatic settings (19, 22, 23), while the role of hormonal therapy has not yet been well established since it is limited to a case series (1).

A recent study analysed 58 patients with recurrent or metastatic uterine AS and 20 with extrauterine AS: most recurrences were abdominal and pelvic and a minor part was distant (29). Relapsed patients were treated mainly with surgery and chemotherapy, alone or in combination. Frequently, chemotherapy regimens were doxorubicin-based or gemcitabine and docetaxel, with a response rate of 40% for the first regimen vs. 25% for the second one. The median OS improved in the group of patients with resection for recurrent lesions. One-third of relapsed patients received hormonal therapy (GnRH agonists, progestins, SERMs and aromatase inhibitors), obtaining a relevant lengthening in OS. Nearly two-thirds of patients with recurrent disease had a high-grade sarcoma, with a median OS of half of the patients with a mixed histology. The Italian Consensus Conference on Management of Uterine Sarcomas in 2020 affirmed that hormonal adjuvant treatment can be offered to patients with uterine AS without sarcomatous overgrowth when hormone receptors are present (11). Expression of estrogen receptors is present in more than two-thirds of patients with this diagnosis, but this positivity is lower in SO in women (10). Carroll described a prolonged response with aromatase inhibitors given initially in an adjuvant setting in a patient with stage IV uterine AS and with a strong staining for estrogen and progesterone receptors, and then for recurrent disease, obtaining a stabilisation of disease and reaching a follow-up of 12.5 years from the diagnosis (15).

## 5 Conclusions

The most important prognostic factors associated with overall survival in our patients diagnosed with uterine adenosarcoma seem to be age and FIGO stage at diagnosis.

## Data availability statement

Dataset are available on request to corresponding author. Requests to access these datasets should be directed to RMan, rosanna.mancari@ifo.it.

## Ethics statement

The studies involving humans were approved by the Institutional Review Board (IRB), and approval was first obtained at the coordinating centre: La Paz University Hospital, 28046 Madrid, Spain (#PI-1382). The studies were conducted in accordance with the local legislation and institutional requirements. Written informed consent for participation was not required from the participants or the participants’ legal guardians/next of kin in accordance with the national legislation and institutional requirements.

## Author contributions

RMan: Writing – review & editing, Writing – original draft, Validation, Supervision, Methodology, Funding acquisition. YY: Writing – review & editing, Validation, Formal analysis. RMac: Writing – review & editing, Resources, Methodology, Data curation. PA: Writing – review & editing, Visualization, Investigation, Data curation. JP: Writing – review & editing, Validation, Software. IS: Writing – review & editing, Software, Formal analysis, Data curation. GC: Writing – review & editing, Validation, Supervision, Conceptualization. EV: Writing – review & editing, Supervision, Funding acquisition. IZ: Writing – review & editing, Writing – original draft, Supervision, Conceptualization.
